# Interaction of PsMYB4 with PsEGL3 inhibits anthocyanin biosynthesis in tree peony yellow flowers

**DOI:** 10.3389/fpls.2025.1595014

**Published:** 2025-06-23

**Authors:** Xiaoning Luo, Sijie Huang, Fangjun Jiang, Qianqian Shi, Yafeng Wen, Mengchen Li, Minhuan Zhang, Yanlong Zhang

**Affiliations:** ^1^ College of Landscape Architecture, Central South University of Forestry and Technology, Hunan, Changsha, China; ^2^ College of Landscape Architecture and Art, Northwest A&F University, Shaanxi, Xianyang, China

**Keywords:** tree peony, yellow flower, anthocyanin biosynthesis, flavonoid pathway, PsMYB4, PsEGL3, negative regulation

## Abstract

Tree peony is a traditional woody flower originating from China and possesses high ornamental value. The yellow cultivar is even more precious, known as the highest grade. However, the molecular mechanism underlying tree peony yellow flower formation is still unclear. Our present work identified two transcription factors (TFs), PsMYB4 and PsEGL3, which were highly expressed in yellow tree peony cultivar. Phylogenetic analysis indicates that PsMYB4 belonged to the R2R3-MYB repressor, while PsEGL3 was clustered into subgroup IIIf of bHLH family. Overexpression of *PsMYB4* and *PsEGL3* respectively in tobacco inhibited anthocyanin synthesis, with *PsMYB4* overexpressing lines being more pronounced and the expression levels of structural genes *NtC4H*, *NtCHS*, *NtCHI*, and *NtDFR* were significantly downregulated, while the expression levels of structural genes in *PsEGL3* transgenic lines showed no significant pattern. On the contrary, the expression of *PsCHS*, *PsCHI*, *PsF3H* and *PsDFR* increased in either *PsMYB4* or *PsEGL3* silencing tree peony petals, in which endogenous *PsMYB4* and *PsEGL3* genes were also inhibited to a certain extent. Yeast two-hybrid (Y2H) and bimolecular fluorescence complementation (BiFC) assays further confirmed PsMYB4 could interact with PsEGL3. Moreover, in dual-luciferase (LUC) assay, PsMYB4 and PsEGL3 synergistically suppressed the promoter activity of *PsCHS*, *PsCHI*, and *PsDFR*, thus inhibiting anthocyanin biosynthesis branch and leading to a metabolic flow towards the flavonol synthesis branch. These findings provide a new perspective for revealing the molecular mechanism of the yellow flower formation in tree peony.

## 1 Introduction

Tree peony (*Paeonia* Section Moutan DC.) is a traditional woody ornamental flower originating from China, renowned as the ‘king of flowers’ due to its grandeur, vibrant colors, and exquisite blooms (Li, 1999). After more than 1,600 years of natural selection and artificial cultivation, tree peony has now formed over 2,000 cultivars worldwide (Yang et al., 2020a). Flower color is a crucial ornamental trait and serves as the classification basis for tree peony, including 9 color systems such as red, pink, purple, yellow, white, black, blue, green, and dual-color. Among them, yellow tree peony is loved by people for its noble and pure image, and its ornamental value is very high. However, currently, there is a widespread phenomenon of impure yellow in the yellow cultivars produced by hybridization (Li et al., 2011). Therefore, it is imperative to carry out molecular breeding research on yellow tree peony cultivars, and exploring the molecular mechanism of yellow flower formation will lay an important theoretical foundation for targeted molecular breeding of yellow tree peony cultivars.

The formation of yellow flowers in plants is mainly determined by the type and content of pigments. Generally, in light yellow flowers, colorless or light yellow flavones and flavonols dominate, while for darker yellow flowers, aurones, chalcones, and carotenoids play a decisive role (An, 1989). Previous studies have shown that flavone, flavonol, and chalcone compounds in yellow tree peony were the main substances responsible for its coloration, including 26 types of flavonoid components such as luteolin glycosides, apigenin glycosides, kaempferol glycosides, and isosalipurposides (Li et al., 2009; Zhou et al., 2011; Shi et al., 2015; Lv et al., 2023). Similarly, for 14 cultivars of yellow tree peony and herbaceous peony, including Itoh hybrid and *Paeonia ludlowii* hybrid, a total of 29 flavonoid compounds, including 28 flavone glycosides and one chalcone glycoside, were isolated and identified through pigment composition analysis (Yang et al., 2020b). Our previous studies have also demonstrated that the pigments responsible for the formation of yellow flowers are flavonoids in ‘High Noon’ (*P. suffruticosa* × *P. lutea*), with tetrahydroxychalcone, apigenin, kaempferol, chrysoetiol, and isorhamnetin dominating, without anthocyanin (Luo et al., 2021).

Flavonoid biosynthesis is primarily governed by three stages of structural genes (*PAL*, *C4H*, *4CL* genes in the first stage; *CHS*, *CHI*, *F3H*, *FNS*, *F3’H*, *FLS* genes in the second stage; *DFR*, *ANS, GT/AT/MT* genes in the third stage), which directly determine the synthesis of different flavonoids such as flavones, flavonols, chalcones, and anthocyanins in plants, thereby affecting flower coloration. Among them, there is a competitive relationship between *FLS* gene and *DFR* gene, which respectively control two flavonoid synthesis branches (flavonol synthesis branch and anthocyanin synthesis branch) (Zhang et al., 2022). The functions of these structural genes have been extensively investigated in a large number of plant species (Winkel-Shirley, 2001). In tree peony, the functions of *PsCHS*, *PsCHI1*, *PsF3’H*, *PsDFR*, *PsANS*, and *PsAOMT* genes were also characterized successively (Zhang et al., 2015; Zhao et al., 2015; Zhang et al., 2018; Zhang et al., 2020; Zhou et al., 2014; Du et al., 2015), and the *DFR* gene could interact with the GST transporter *PsGSTF3* gene responsible for transporting anthocyanins to vacuoles, which together promote the coloration of tree peony petals (Han et al., 2022). It is worth noting that the up-regulation of *PsFLS* gene and down-regulation of *PsDFR* gene promote the metabolic flow of flavonoid synthesis pathway toward flavonol synthesis, thus indirectly hindering anthocyanin synthesis, which is the genetic basis for the formation of yellow tree peony flowers (Luo et al., 2021; Zhang et al., 2022).

Extensive research has been conducted on transcription factors (TFs) that govern the expression intensity and pattern of structural genes associated with flavonoid synthesis. Certain members of the R2R3-MYB and bHLH families, along with select WD40 proteins, modulate the expression of flavonoid biosynthesis genes in the form of MYB-bHLH-WD40 (MBW) complex (Ding et al., 2020; Schwinn et al., 2006; Hsu et al., 2015). However, only R2R3-MYB and bHLH TFs possess DNA-binding capabilities and exhibit specific binding to the promoters of structural genes in flavonoid biosynthesis pathway, whereas WD40 proteins typically assume a stabilizing role within the MBW complex (Van Nocker and Ludwig, 2003; Hichri et al., 2011). The R2R3-MYB TFs can be categorized into 28 subgroups based on the variations of conserved motifs in the C-terminal variable region (Dubos et al., 2010). Among them, subgroup 4 (SG4) inhibits anthocyanin synthesis, such as MYB4 in *Arabidopsis* (Wang et al., 2020), MYB182 in *Populus* (Yoshida et al., 2015), MdMYB16 in *Malus domestica* (Xu et al., 2017), PhMYB27 in *Petunia hybrida* (Albert et al., 2014), MYB4a/4b and MYBC2-L1/L2/L3 in *Vitis vinifera* (Cavallini et al., 2015; Pérez-Díaz et al., 2016). At present, the MYB inhibitors preliminarily identified in tree peony include PoMYB2 in *P. ostii* and PqMYB4 isolated from the leaves of *P. qiui* (Gao et al., 2016; Huo et al., 2020). SG7 promotes the synthesis of flavonols, such as GhMYB1 in *Gerbera hybrida* (Zhong et al., 2020). Moreover, in *P. rockii* ‘Shu Sheng Peng Mo’, SG7 member PrMYBa1 contributes to the petal blotch formation by interacting with PrMYBa2 to activate *PrF3H* gene (Zhu et al., 2023). In our previous study, we also found that SG7 member PsMYB111 promotes metabolic flow toward flavonol synthesis in *P. suffruticosa* ‘High Noon’ petals by independently regulating the expression of *PsCHS* and *PsFLS*, ultimately participating in the formation of yellow tree peony flowers (Luo et al., 2021). In current studies, it has been identified that bHLH TFs participated in the regulation of plant flavonoid metabolism belong to the IIIf subgroup, most of which are known to interact with MYBs to promote anthocyanin biosynthesis such as DhbHLH1 in *Dendrobium hybrid*s and bHLH3 in *Fructus Mori* (Li et al., 2017; Li H. et al., 2020). In *P. suffruticosa* ‘Qing Hai Hu Yin Bo’, PsMYB12, along with bHLH and WD40, plays a pivotal role in the development of petal blotch by regulating the organ-specific expression of *PsCHS* in the form of MBW complex (Gu et al., 2018). PsMYB58, as a positive regulator of anthocyanin biosynthesis in *P. suffruticosa* ‘Er Qiao’, functions by interacting with PsbHLH1 and PsbHLH3 (Zhang et al., 2021). However, some bHLH TFs in subgroup IIIf have recently been reported as repressors of anthocyanin biosynthesis, including CpbHLH1 from *Chimonanthus praecox* (Zhao et al., 2020), SmbHLH1 from *Solanum melongena* (Duan et al., 2021), HLH4 from *Arabidopsis thaliana* (Hou et al., 2022), and FtTT8 from *Fagopyrum tataricum* (Deng et al., 2023). To date, it is still unclear how MYB inhibitors and related bHLH TFs participate in regulating the formation of yellow tree peony flowers, which provides us with a novel approach to study their molecular regulatory mechanism.

In our previous research, based on transcriptome analysis of tree peony cultivars ‘High Noon’ and ‘Roufurong’, we identified that PsMYB4 and PsEGL3 TFs may be involved in regulating the formation of tree peony yellow flowers, but their molecular mechanisms are still unclear (Luo et al., 2021). In this study, the potential regulatory mechanism of yellow flower formation in tree peony was discussed. Through pigment detection, gene expression analysis, subcellular localization, gene overexpression experiments, virus-induced gene silencing (VIGS) analyses, yeast two-hybrid (Y2H) assays, bimolecular fluorescence complementation (BiFC) assays, and dual-luciferase (LUC) transient expression assays, we have confirmed PsMYB4 and PsEGL3 as negative regulators of anthocyanin biosynthesis, which exert an influence on the formation of yellow flowers in tree peony. These findings offer a novel perspective on the regulation of tree peony yellow flowers and have potential implications for the breeding of tree peony as well as other flowering plants with a broader spectrum of floral colors.

## 2 Materials and methods

### 2.1 Plant materials

**Figure 1:**
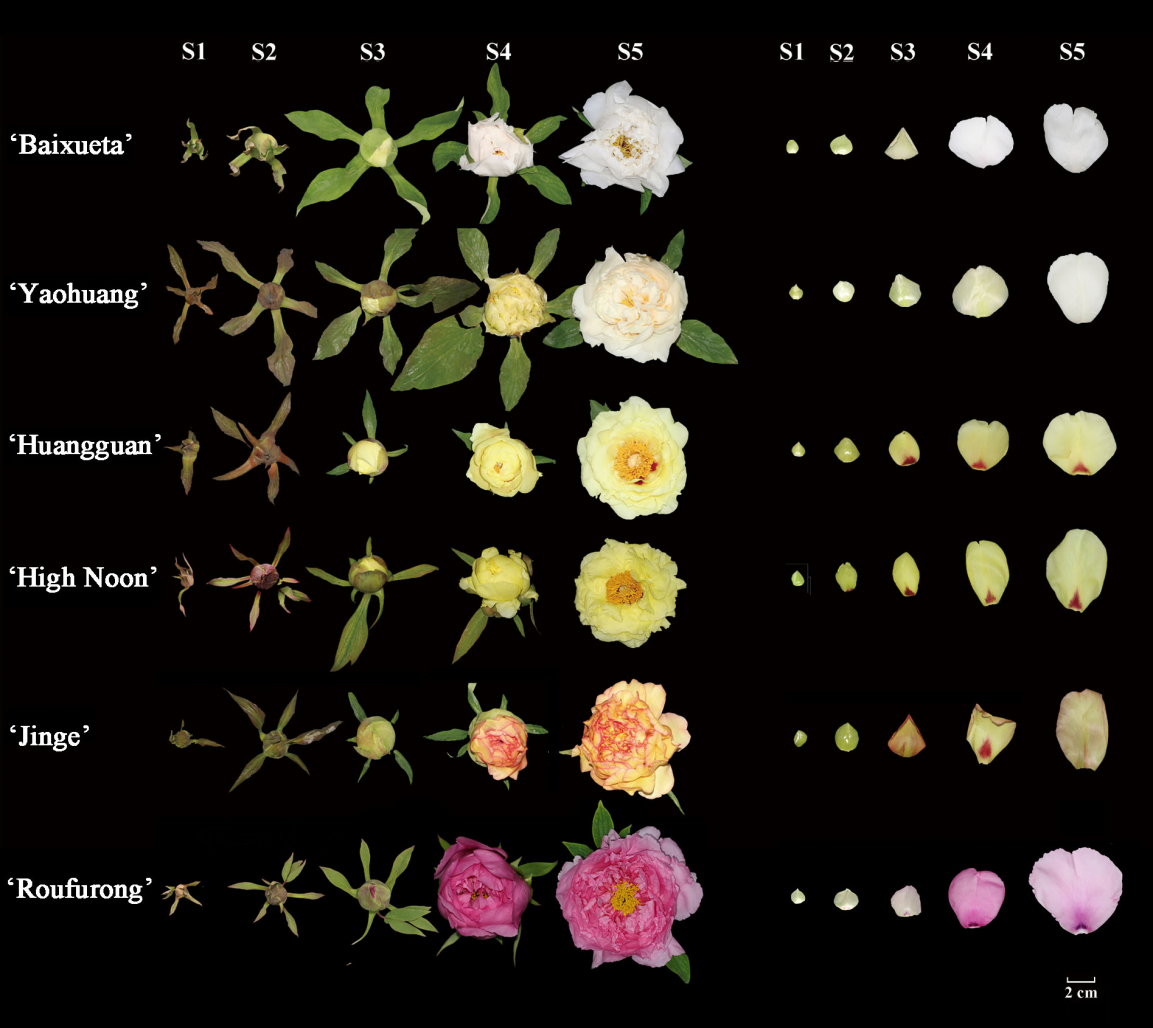
Floral phenotypes of tree peony cultivars at five blooming stages. Stage 1 (S1): unpigmented compact bud; Stage 2 (S2): slightly pigmented soft bud; Stage 3 (S3): slightly pigmented flower about to bloom; Stage 4 (S4): pigmented blooming flower; Stage 5 (S5): pigmented fully blooming flower with exposed anthers.

The tree peony cultivars ‘Baixueta’, ‘Yaohuang’, ‘Huangguan’, ‘High Noon’, ‘Jinge’, and ‘Roufurong’ were cultivated under adequate conditions of light and moisture in the Tree Peony Garden at Northwest A&F University, Shaanxi Province, China (**Figure 1**). The middle petal samples from 9 plants of each cultivar were collected at five stages defined according to a previous method (S1, unpigmented compact bud; S2, slightly pigmented soft bud; S3, slightly pigmented flower about to bloom; S4, pigmented blooming flower; S5, pigmented fully blooming flower with exposed anthers) (Zhou et al., 2011), of which spots of ‘Huangguan’, ‘High Noon’, ‘Jinge’, and ‘Roufurong’ were removed. *N. tabacum* and *N. benthamiana* plants were cultivated under controlled conditions in a programmable incubator, maintaining a 16 h light/8 h dark photoperiod at 25°C. Color-related values (L*, a*, b*, C*, and *h*) of fresh petals were quantified using a tristimulus color meter (CR-400, Konica Minolta, Osaka, Japan). The petals for the remaining tests were promptly frozen in liquid nitrogen, and subsequently stored at -80°C.

### 2.2 Measurement of flavonoids

The frozen petals of four tree peony cultivars were quickly pulverized into a fine powder using liquid nitrogen. Approximately 0.3 g of petal powder was dissolved in a solution of methanol-hydrochloric acid (99: 1, V/V) (for anthocyanins) and a separate solution of methanol (for other flavonoids) (6 ml each), respectively. Next, the samples were extracted at 4°C for 24 h in the dark, and agitated once every 6 h. The supernatant of the samples was collected following centrifugation at 10,000 r/min for 10 minutes. The extraction solution was scanned with the full wavelength of 200~850 nm by Multiskan Spectrum (SP-Max 2300A2, Shanghai, China), and the maximum absorption value at about 520 nm was taken to calculate the total anthocyanin concentration. Each sample was subjected to three biological replicates. The content of other flavonoid components was assessed by HPLC (LC-2030C 3D, Shimadzu, Kyoto, Japan) system equipped with a 4.6 × 250 mm C18 column and a diode array detector (Shimadzu, Kyoto, Japan). Mobile phases comprised of eluent A, a 0.04% aqueous solution of formic acid, and eluent B, acetonitrile. The gradient progressed as follows: 5% B (0 min), 40% B (40 min), 100% B (45 min), 100% B (55 min), 5% B (60 min). The flow rate was maintained at 0.5 ml/min while the column temperature was set to 40°C. The mean values and SDs were determined based on three replicates per sample.

### 2.3 Expression analysis by qRT-PCR

The total RNA from petals at S1-S5 in each tree peony cultivar was extracted using Plant RNA Kit (Tiangen, Beijing, China). First-strand cDNA was synthesized utilizing the PrimeScript™ RT Master Mix reverse transcription kit (Takara, Dalian, China). Subsequently, qPCR was conducted using TB Green TaKaRa Premix Ex Taq™ II (TaKaRa, Dalian, China). The conditions for amplification entailed one cycle at 95°C for 15 s, followed by 45 cycles comprising 95°C for 5 s, 58°C for 30 s, and 72°C for 31 s. Specific primers are detailed in **Supplementary Table S1**. *Psubiquitin* gene was used as an internal reference for the expression level normalization by employing the 2^−ΔΔCT^ method (Livak and Schmittgen, 2001). Three biological replicates were independently conducted for each qPCR assay.

### 2.4 Sequence and subcellular localization analyses

Phylogenetic trees of *PsMYB4* and *PsEGL3* were constructed from evolutionary distance data with Neighbor-joining method by MEGA 6.0. The gene names and login numbers used in phylogenetic trees were shown in **Supplementary Tables S2, S3**. The open reading frames (ORFs) of *PsMYB4* and *PsEGL3*, lacking the termination codon, were individually inserted into pCAMBIA1302-GFP vectors. Next, the resulting constructs were transformed into the competent *Agrobacterium* strain GV3101 (Yan et al., 2012). *Agrobacterium* cultures transformed by each construct were resuspended in infiltration buffer containing 10 mM MES, 100 mM AS and 10 mM MgCl_2_, after which they were injected into leaves of *N. benthamiana*. Three days after injection, the GFP fluorescence signal was observed using a Nikon C2-ER confocal laser scanning microscope (Nikon, Tokyo, Japan). The experiment was conducted in triplicate. The primers are shown in **Supplementary Table S4**.

### 2.5 Stable overexpression in tobacco

For gene functional analysis, the recombinant pCAMBIA1302 vectors carrying *PsMYB4* and *PsEGL3* were transferred into *Agrobacterium* GV3101, respectively. The leaf discs of *N. tabacum* were utilized as for transgenic transformation, following a previously described methodology (Horsch et al., 1985). Subsequently, the plantlets were transplanted into soil, subsequently cultivated in a controlled greenhouse environment until blooming. The transgenic lines were validated through qRT-PCR using specific downstream primers targeting *PsMYB4* and *PsEGL3*, along with a specific upstream primer derived from the *CaMV35S* promoter. The petals of T2 transgenic plants were collected for the quantification of color-related values, including L*, a*, C*, and *h*. Meanwhile, the flavonoid contents were determined as described in the section HPLC analysis, while the structural genes were detected using qRT-PCR. All specific primers are listed in **Supplementary Table S5**.

### 2.6 Virus-induced gene silencing in tree peony

The TRV1 and TRV2 vectors were employed to perform VIGS experiments in *P. suffruticosa* ‘High Noon’ for the purpose of characterizing the functions of *PsMYB4* and *PsEGL3*. *PsMYB4* and *PsEGL3* fragments measuring 203 and 318 bp were inserted into the TRV2 vectors using PstI and Xhol, respectively. Target fragments and linear TRV2 vectors were treated with T4 DNA ligase to generate TRV2-*PsMYB4* and TRV2-*PsEGL3*. Recombinant vectors and empty vectors of TRV1 and TRV2 were introduced into *Agrobacterium* strain GV3101, resuspended in an infiltration buffer with 10 mM MES, 100 mM AS and 10 mM MgCl_2_, and incubated at room temperature for 2 h. *Agrobacterium* strain GV3101 cells containing TRV1 mixed with TRV2 (CK), TRV1 mixed with TRV2-*PsMYB4*, TRV1 mixed with TRV2-*PsEGL3* at a 1:1 ratio were injected into the buds of ‘High Noon’ at S2 stage. The injection volume of each combination was 1 ml, and the injection should be gentle and slow to ensure that the suspension evenly penetrates into the flower buds. The injection was repeated two days later. The petal samples (spots removed) were collected after blooming to detect the flavonoid contents as described in the section HPLC analysis. In addition, qRT-PCR was performed to determine gene expression levels. Specific primers utilized in this study are documented in **Supplementary Table S6**.

### 2.7 Yeast two-hybrid assay

To test the yeast two-hybrid between PsMYB4 and PsEGL3, the ORF of *PsMYB4* was fused into pGADT7 AD vector by homologous recombination to generate pGADT7-PsMYB4 recombinant vector. Simultaneously, the ORF of *PsEGL3* was fused into pGBKT7 BD vector to generate pGBKT7-PsEGL3 recombinant vector. Subsequently, pGADT7-PsMYB4 and pGBKT7-PsEGL3, pGADT7 empty vector and pGBKT7-PsEGL3 (negative control), as well as pGBKT7–53 and pGADT7-T (positive control) were co-transformed into AH109 yeast competent cells, respectively. After confirming the absence of any self-activation activity, yeast cells successfully co-transformed were grown on SD/-Trp/-Leu, SD/-Trp/-Leu/-His, SD/-Trp/-Leu/-His/-Ade adding X-α-Gal screening medium respectively. The specific primers utilized are documented in **Supplementary Table S7**.

### 2.8 Bimolecular fluorescence complementation assay

The ORFs of *PsMYB4* and *PsEGL3* lacking the termination codon were PCR-amplified. *PsMYB4* fragment was fused into pUC-SPYNE vector to generate PsMYB4-nYFP, and *PsEGL3* fragment was fused into pUC-SPYCE vector to generate PsEGL3-cYFP using BamHI and Xhol restriction sites. The recombinant vectors were co-transformed transiently into *N. benthamiana* leaves as described by previous study (Hellens et al., 2005). BZIP63-nYFP co-transformed with BZIP63-cYFP was the positive control. The negative controls consisted of PsMYB4-nYFP co-transformed with 35S-cYFP and PsEGL3-cYFP co-transformed with 35S-nYFP. The YFP fluorescence signal was visualized 72 h after injection. The experiment was conducted in triplicate. All primers utilized in this study are shown in **Supplementary Table S8**.

### 2.9 Luciferase reporter assay

The promoters of *PsCHS, PsCHI* and *PsDFR* were cloned from genomic DNA of ‘High Noon’ petals based on previous genome DNA sequences (China National Gene Bank, CNP0003098) and transcriptome cDNA sequences (Genome Sequence Archive, CRA005005) of tree peony. The analysis and prediction of cis-elements in promoters were conducted using online website PlantCARE (http://bioinformatics.psb.ugent.be/webtools/plantcare/html). The *PsMYB4* and *PsEGL3* ORFs were cloned into pCAMBIA1302 vectors as effectors, while the promoters of *PsCHS*, *PsCHI*, and *PsDFR* were cloned into pGreenII0800-LUC vectors as reporters. All effectors and reporters were transformed into *Agrobacterium* strain GV3101 and subsequently co-transformed into *N. benthamiana* leaves according to the method of previous study (Hellens et al., 2005). The empty pCAMBIA1302 vectors co-transformed with different LUC recombination vectors were regarded as negative controls. At 48 h post-infiltration, the enzyme activities of LUC and REN were assessed using a dual-luciferase assay system on GloMax^®^ Discover (Promega, Madison, USA). The LUC/REN ratio determined the activity, and three biological replicates were conducted per assay. All primers utilized in the present study are documented in **Supplementary Table S9**.

## 3 Results

### 3.1 Flavonoids in different tree peony cultivars

**Figure 2:**
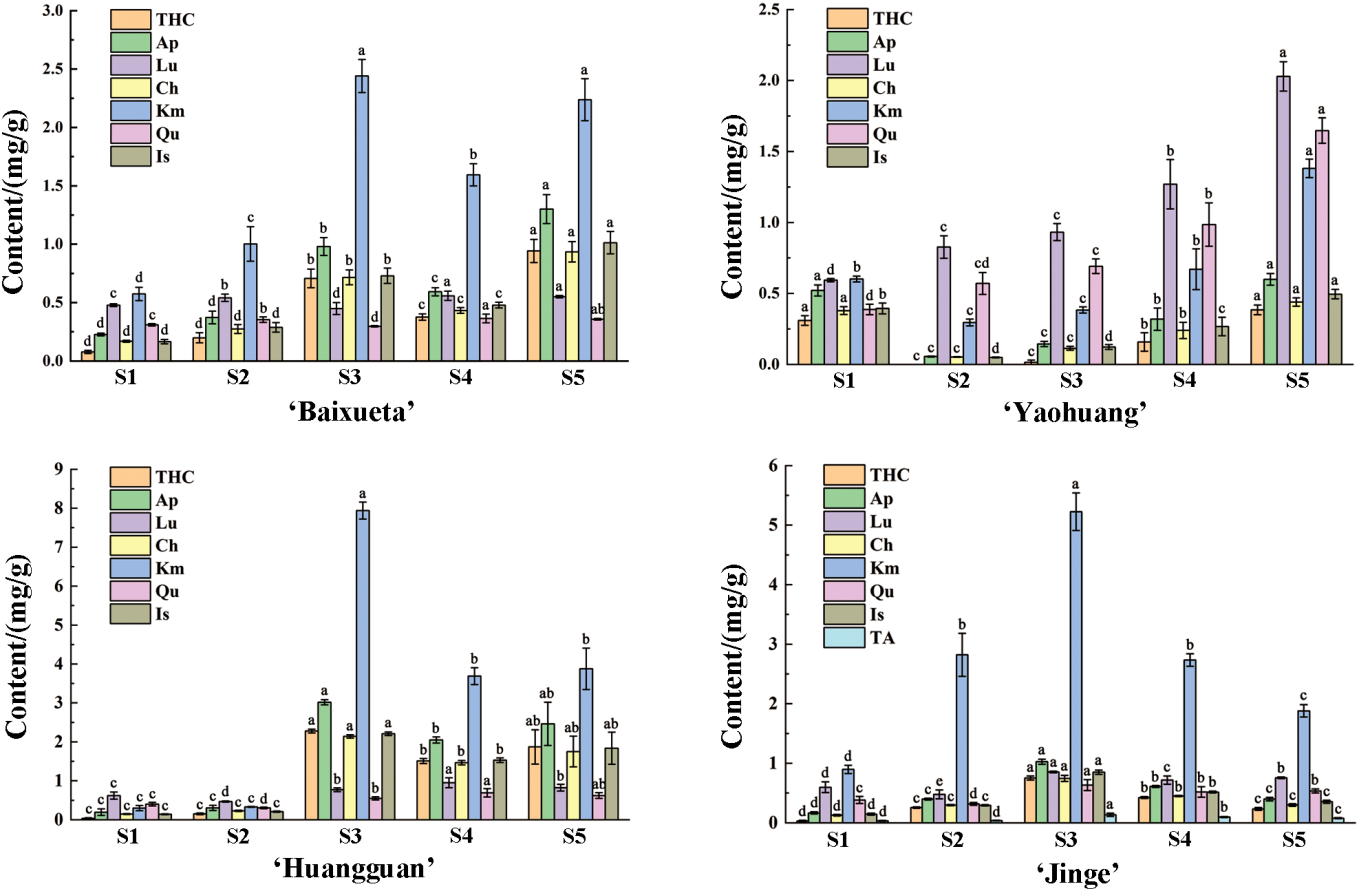
Flavonoid contents of tree peony cultivars at five blooming stages. Different letters (a-d) signify statistically significant differences (p<0.05) among various flavonoids by Duncan’s test. THC, tetrahydroxychalcone; Ap, apigenin; Lu, luteolin; Ch, chrysoeriol; Km, kaempferol; Qu, quercetin; Is, isorhamnetin; TA, total anthocyanins.

The flavonoid content and composition of the tree peony petals of yellow cultivar ‘High Noon’ and purple red cultivar ‘Roufurong’ were characterized in a previous study (Luo et al., 2021). Thus, the present study employed high-performance liquid chromatography (HPLC) experiments in the other four cultivars ‘Baixueta’ (white), ‘Yaohuang’ (yellowish), ‘Huangguan’ (light yellow), ‘Jinge’ (reddish yellow) at five blooming stages. As a result, no anthocyanins were detected in ‘Baixueta’, ‘Yaohuang’, ‘Huangguan’ and ‘High Noon’ except for ‘Jinge’ and ‘Roufurong’, which was consistent with their floral color phenotype (**Figure 2**). Our previous research has shown that ‘High Noon’ has the highest tetrahydroxychalcone (THC) content and co-dominates the formation of its yellow flowers with rich flavones and flavonols such as kaempferol (Km), apigenin (Ap), chrysoeriol (Ch) and isorhamnetin (Is) (Luo et al., 2021). However, the contents of flavones and flavonols in ‘Baixueta’, ‘Yaohuang’, ‘Huangguan’ and ‘Jinge’ were much higher than that of THC, and the contents of various flavonoids in ‘Baixueta’ and ‘Yaohuang’ were obviously lower than those in ‘Huangguan’ and ‘Jinge’ (**Figure 2**), which may be the reason for their lighter flower color. Among them, the content of Km, one of the flavonols in ‘Baixueta’, ‘Huangguan’ and ‘Jinge’ was obviously higher than that of other flavonoids, and its change trend was consistent in ‘Baixueta’ and ‘Huangguan’. In regard to ‘Jinge’, the content of Km exhibited an initial upward trend followed by a subsequent decline. It is noteworthy that anthocyanin was detected in ‘Jinge’, and the total anthocyanin content increased significantly at S3 and then decreased gradually at S4 and S5. This is consistent with the simultaneous red and yellow phenotype of ‘Jinge’ petals from S3 to S5, during which the red color gradually decreases.

### 3.2 Expression patterns of *PsMYB4* and *PsEGL3* in different tree peony cultivars

**Figure 3:**
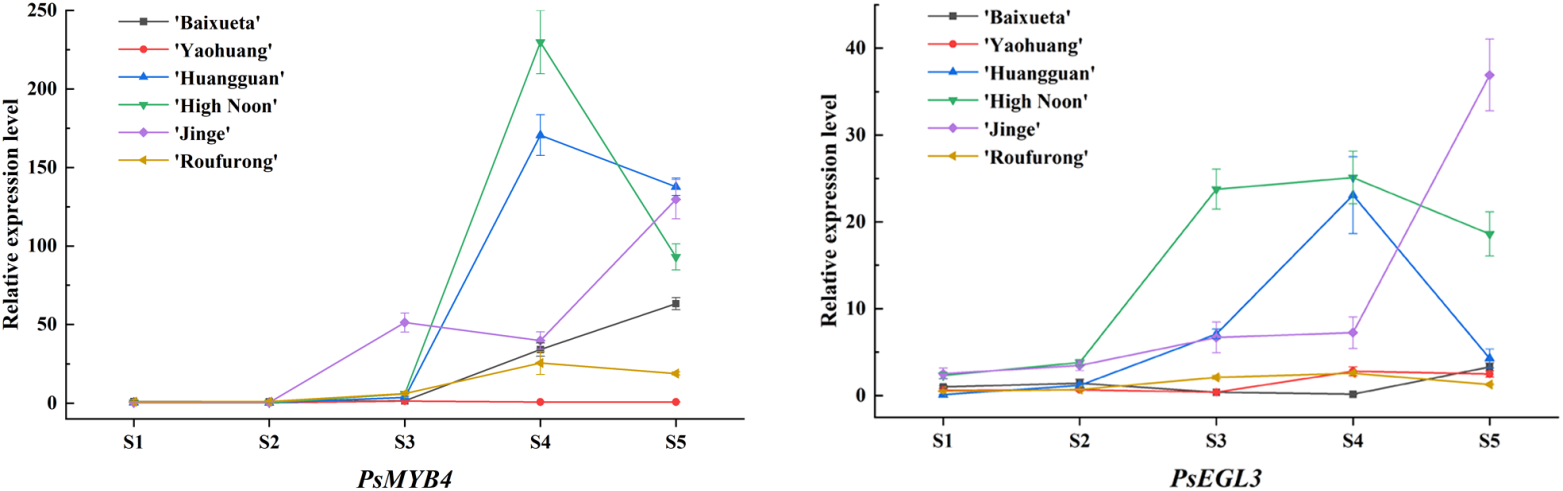
Expression pattern analysis of *PsMYB4* and *PsEGL3* at five stages in the petals of six tree peony cultivars.

To investigate the potential functions of *PsMYB4* and *PsEGL3* in yellow tree peony flowers, we conducted qRT-PCR analysis to examine the expression patterns of *PsMYB4* and *PsEGL3* at five different blooming stages across six tree peony cultivars. The results are presented in **Figure 3**, and the expression patterns of *PsMYB4* and *PsEGL3* exhibited a high degree of consistency across all six tree peony cultivars. Overall, their expression levels were higher in ‘High Noon’, ‘Huangguan’ and ‘Jinge’, while lower in ‘Baixueta’, ‘Yaohuang’ and ‘Roufurong’. It indicated that the expression levels of *PsMYB4* and *PsEGL3* were higher in the darker yellow cultivars, while lower in the purple red, white and light yellow cultivars. Moreover, the expression levels of *PsMYB4* and *PsEGL3* in ‘High Noon’ and ‘Huangguan’ showed an accordant trend of first increasing and then decreasing, reaching their maximum values at S4. Nevertheless, the expression of *PsMYB4* in ‘Jinge’ exhibited a pattern of initial increase, followed by decrease, and subsequent re-increase, reaching its peak at S5, while the expression pattern of *PsEGL3* in ‘Jinge’ showed a continuous upward trend, reaching a peak rapidly at S5, which may be related to the presence of anthocyanins in the petals of ‘Jinge’. These results suggested that *PsMYB4* and *PsEGL3* may inhibit the synthesis of anthocyanins in tree peony yellow flowers.

### 3.3 Cluster analysis and subcellular localization of *PsMYB4* and *PsEGL3*

**Figure 4:**
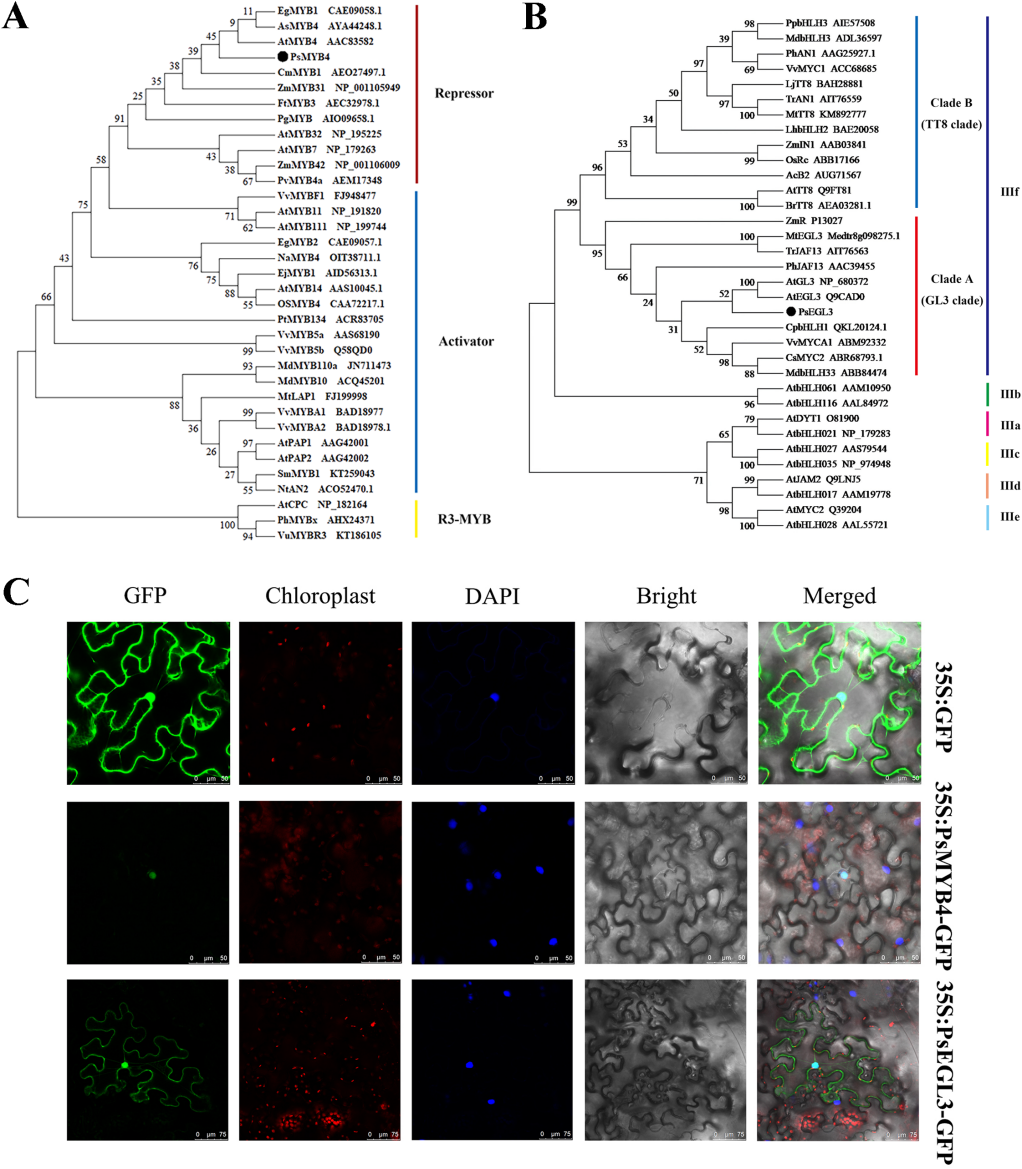
Phylogenetic tree analysis and subcellular localization of PsMYB4 and PsEGL3. **(A)** Phylogenetic tree analysis of PsMYB4 with related MYB proteins in other plant species. **(B)** Phylogenetic tree analysis of PsEGL3 with related bHLH proteins in other plant species. **(C)** Subcellular localization of PsMYB4 and PsEGL3 in epidermal cells of tobacco leaves. GFP, GFP fluorescence; Chloroplast, chloroplast fluorescence; DAPI, DAPI fluorescence; Bright, bright field; Merged, superposition of bright field and fluorescence. Bars, 50 and 75 µm.

In the preliminary research, the full-length sequences of *PsMYB4* and *PsEGL3* genes were isolated, and a homologous alignment revealed that the N-terminus of *PsMYB4* contained a conserved R2R3 domain and a bHLH-interacting motif, while its C-terminus also contained C1 and C2 inhibitory motifs (Luo et al., 2021). Similarly, the amino acid sequence of PsEGL3 consisted of a bHLH domain and an N-terminal MYB-interacting region (MIR) (Luo et al., 2021). The MIR of subgroup IIIf bHLH proteins is pivotal for binding to MYB TFs. We further performed cluster analysis on PsMYB4 and other homologous proteins involved in flavonoid biosynthesis, and found that PsMYB4 belonged to the R2R3-MYB repressor and had the closest relationship with SG4 member AtMYB4 in *Arabidopsis* (**Figure 4A**). Another phylogenetic tree, constructed using bHLH TFs derived from other species that involved in the regulation of anthocyanin synthesis, showed that PsEGL3 clustered into the GL3 clade of subgroup IIIf with high similarity to AtGL3, AtEGL3, CpbHLH1, VvMYCA1, CsMYC2, MdbHLH33 (**Figure 4B**).

To investigate the subcellular localization of PsMYB4 and PsEGL3, the fusion proteins 35S:PsMYB4-GFP and 35S:PsEGL3-GFP were transiently expressed in six-week-old *Nicotiana benthamiana* leaves, while the protein 35S:GFP served as a negative control. The subcellular localization of proteins was observed after 72h. The 35S:GFP protein distributed extensively in cytoplasm, especially in the cell membrane and nucleus (**Figure 4C**). Whereas the protein 35S:PsMYB4-GFP was exclusively localized in the nucleus, and the protein 35S:PsEGL3-GFP was primarily detected in the nucleus, suggesting that PsMYB4 and PsEGL3 are nuclear proteins (**Figure 4C**).

### 3.4 Overexpression of *PsMYB4* and *PsEGL3* inhibits anthocyanin biosynthesis in tobacco

**Figure 5:**
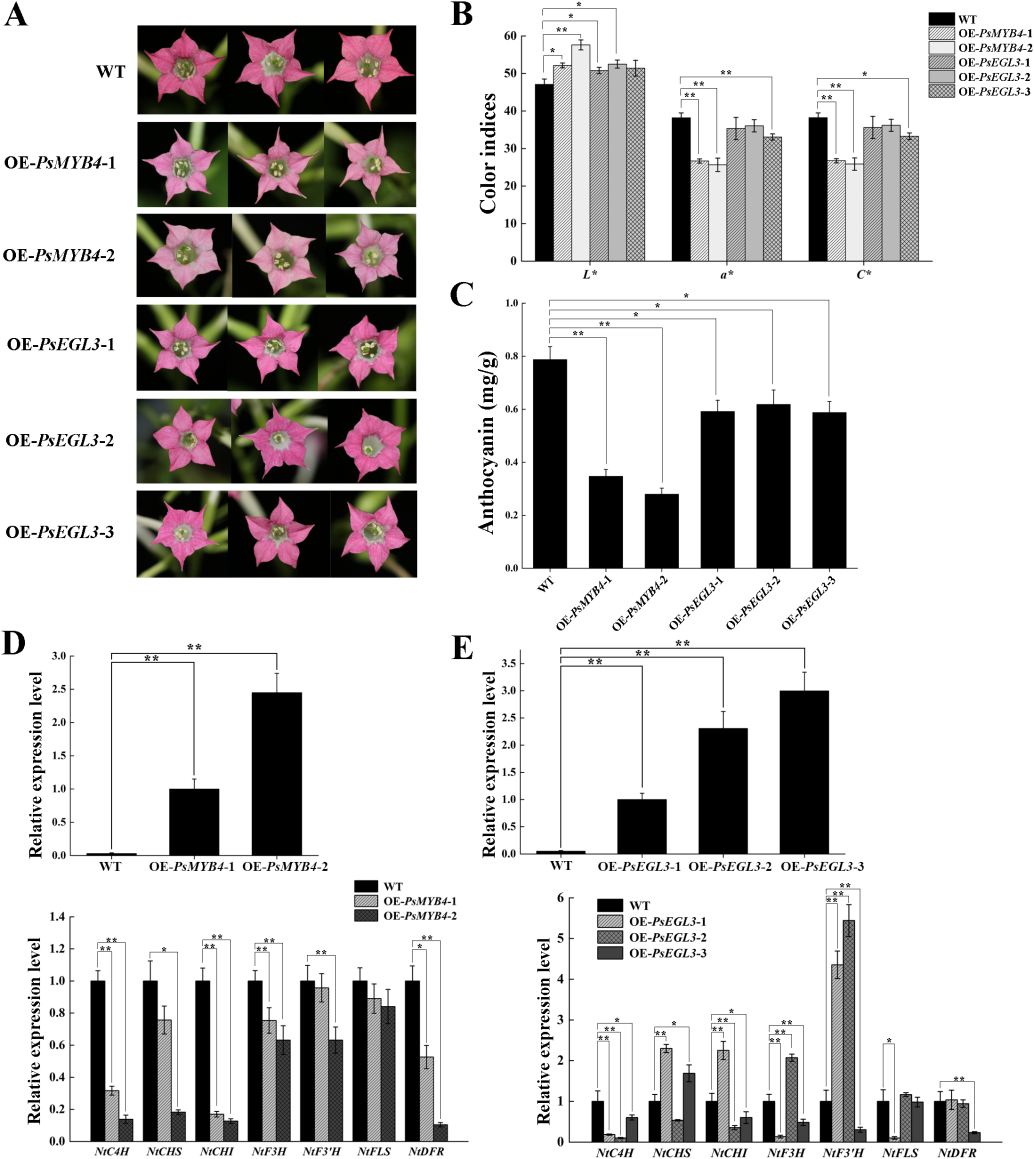
Functional verification of *PsMYB4* and *PsEGL3* in tobacco. **(A)** Flower phenotypes in wild-type (WT) and transgenic tobacco plants. **(B)** Color indices of petals from WT and transgenic tobacco plants. **(C)** Anthocyanin contents in petals of WT and transgenic tobacco plants. **(D, E)** Expression patterns of endogenous TF genes (*NtMYB4* and *NtbHLH*) and flavonoid biosynthetic genes (*NtC4H*, *NtCHS*, *NtCHI*, *NtF3H*, *NtF3’H*, *NtFLS*, *NtDFR*) in petals of WT and transgenic tobacco plants. L* represents the brightness. a* represents the redness. C* represents chroma. All data are means ± SDs from three biological replicates. **p<0.01, *p<0.05.

To validate the function of *PsMYB4* and *PsEGL3* in flavonoid biosynthesis, we generated transgenic tobacco lines overexpressing *PsMYB4* and *PsEGL3*. Two *PsMYB4* transgenic tobacco lines and three *PsEGL3* transgenic tobacco lines were ultimately obtained, named OE-*PsMYB4*-1/2, and OE-*PsEGL3*-1/2/3, respectively. Compared to the deep pink color of wild type tobacco, the corolla color of OE-*PsMYB4–*1 and OE-*PsMYB4–*2 lines was significantly lighter, while the corolla color of OE-*PsEGL3*-1, OE-*PsEGL3–*2 and OE-*PsEGL3–*3 lines was slightly lighter (**Figure 5A**). Next, the floral color values of transgenic plants were determined, and the results indicated a significant decrease in the a* and C* values of OE-*PsMYB4–*1 and OE-*PsMYB4–*2 compared to the wild-type, while the L* values of OE-*PsMYB4–*1 and OE-*PsMYB4–*2 were notably higher than that of the wild-type (**Figure 5B**). The color values of three *PsEGL3* transgenic lines were not consistent, and the L* values increased significantly in OE-*PsEGL3–*1 and OE-*PsEGL3*-2. The L* value in OE-*PsEGL3–*3 had no significant change, but the a* value and C* value decreased significantly. In contrast, the a* and C* values of OE-*PsEGL3–*1 and OE-*PsEGL3–*2 changed inapparently (**Figure 5B**). In accordance with floral color phenotype, the anthocyanin content in the petals of two *PsMYB4* transgenic strains was significantly lower than that of the wild-type at p<0.01, whereas the anthocyanin content of three *PsEGL3* transgenic strains exhibited a significant decrease compared to the wild-type at p<0.05 (**Figure 5C**).

Furthermore, the gene expression analysis confirmed that the overexpression of *PsMYB4* in tobacco resulted in varying degrees of down-regulation of several structural genes, including *NtC4H*, *NtCHS*, *NtCHI*, *NtF3H*, *NtF3’H*, *NtFLS* and *NtDFR*. Notably, significant down-regulation was observed for *NtC4H*, *NtCHS*, *NtCHI* and *NtDFR* in the OE-*PsMYB4–*2 transgenic lines (**Figure 5D**). The results are consistent with the analysis of anthocyanin content and floral color phenotype, suggesting that PsMYB4 may exert inhibitory effects on anthocyanin biosynthesis by down-regulating the expression of structural genes involved in flavonoid biosynthesis, particularly *NtC4H*, *NtCHS*, *NtCHI*, and *NtDFR*. Compared to the wild-type, the expression levels of *NtCHS*, *NtCHI*, *NtF3H*, *NtF3’H*, *NtFLS* and *NtDFR* exhibited inconsistency in three *PsEGL3* transgenic tobacco lines, however, down-regulation of *NtC4H* was observed across all three transgenic lines (**Figure 5E**). These results indicates that PsEGL3 may not play a major inhibitory role on anthocyanin biosynthesis, but assists PsMYB4 in regulation.

### 3.5 Silencing of *PsMYB4* and *PsEGL3* affects the formation of tree peony yellow flowers

**Figure 6:**
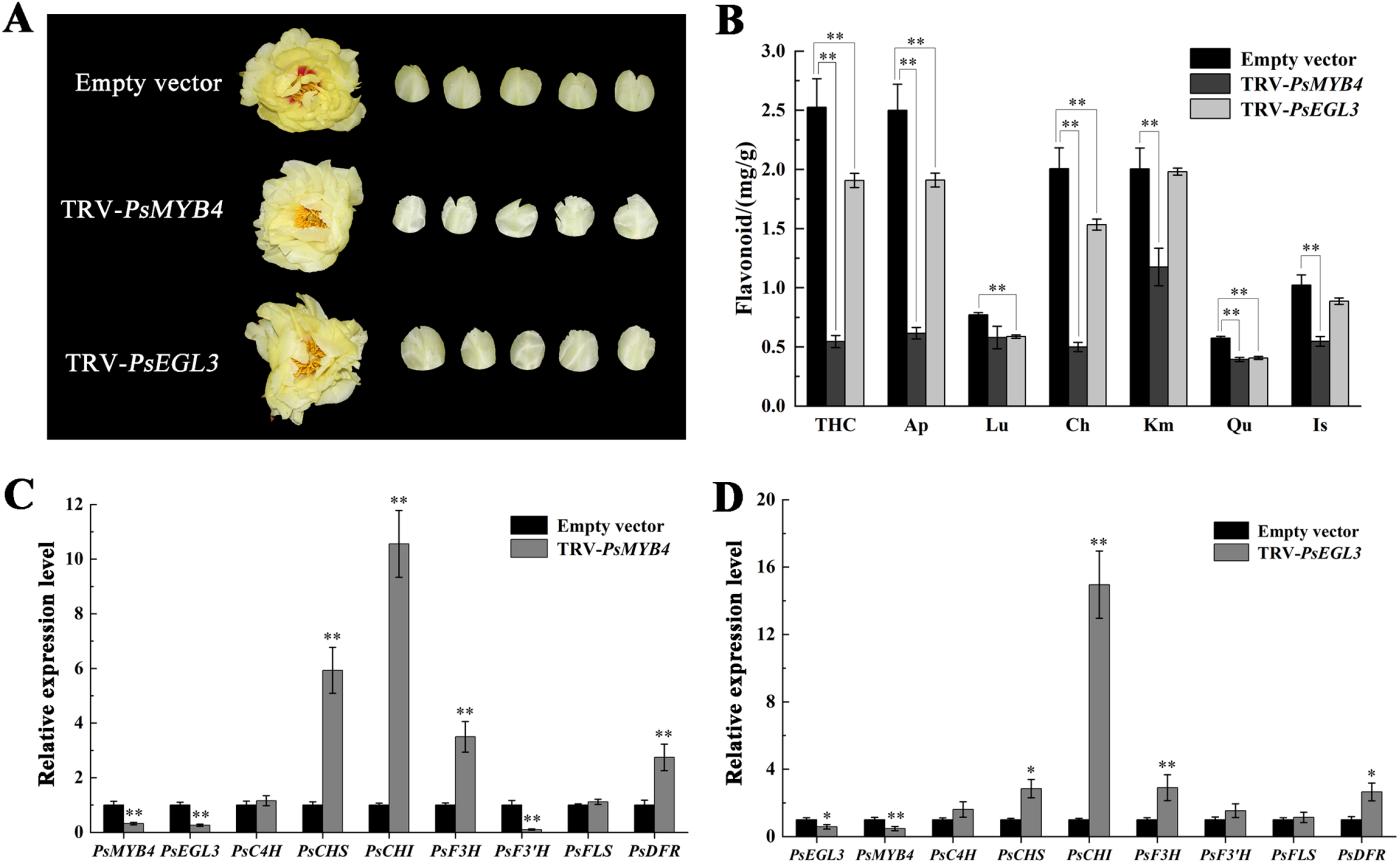
Functional verification of *PsMYB4* and *PsEGL3* in tree peony cultivar ‘High Noon’. **(A)** Flower phenotypes of ‘High Noon’ bearing a TRV empty vector, TRV-*PsMYB4* and TRV-*PsEGL3*, respectively. **(B)** Flavonoid contents in flowers of ‘High Noon’ bearing a TRV empty vector, TRV-*PsMYB4* and TRV-*PsEGL3*, respectively. **(C)** Expression patterns of *PsMYB4, PsEGL3* and flavonoid biosynthetic genes (*PsC4H*, *PsCHS*, *PsCHI*, *PsF3H*, *PsF3’H*, *PsFLS*, *PsDFR*) in petals of ‘High Noon’ bearing a TRV empty vector and TRV-*PsMYB4*, respectively. **(D)** Expression patterns of *PsEGL3, PsMYB4* and flavonoid biosynthetic genes (*PsC4H*, *PsCHS*, *PsCHI*, *PsF3H*, *PsF3’H*, *PsFLS*, *PsDFR*) in petals of ‘High Noon’ bearing a TRV empty vector and TRV-*PsEGL3*, respectively. All data are means ± SDs from three biological replicates. **p<0.01, *p<0.05.

To further investigate the function of *PsMYB4* and *PsEGL3* in tree peony yellow flowers, the transcription of *PsMYB4* and *PsEGL3* was disturbed by VIGS system. Finally, we obtained TRV-*PsMYB4* and TRV-*PsEGL3* tree peony lines, with TRV1 and TRV2 empty vectors injected lines as a control. Next, yellow petals with their spots removed were collected for further analysis. As shown in **Figure 6A**, the yellow petals of TRV-*PsMYB4* and TRV-*PsEGL3* lines were lightened, notably in TRV-*PsMYB4* line. The contents of flavonoids such as THC, Ap, Ch, Km, Qu and Is in TRV-*PsMYB4* line decreased significantly, while the contents of THC, Ap, Lu, Ch and Qu in TRV-*PsEGL3* line decreased significantly (**Figure 6B**), consistent with their floral color phenotypes.

The expression of flavonoid biosynthesis-related genes in silenced petals was determined using qRT-PCR methods. As a result, the expression of *PsMYB4* in TRV-*PsMYB4* petals was down-regulated by 68% compared to the control, while *PsEGL3* in TRV-*PsEGL3* petals showed a 42% decrease, confirming successful gene silencing in tree peony petals (**Figures 6C, D**). When *PsMYB4* was silenced, the expression level of *PsEGL3* was largely inhibited by 74%. Similarly, in TRV-*PsEGL3* petals, the expression level of *PsMYB4* was also decreased by 53%. It indicates that there may be an interaction relationship between *PsMYB4* and *PsEGL3*. The expression of four key flavonoid biosynthesis-related genes (*PsCHS*, *PsCHI*, *PsF3H* and *PsDFR*) was remarkably increased in TRV-*PsMYB4* petals compared to the control line (**Figure 6C**). The expression of *PsCHS*, *PsCHI*, *PsF3H* and *PsDFR* was also increased in TRV-*PsEGL3* petals (**Figure 6D**). Therefore, we speculate that PsMYB4 and PsEGL3 may synergistically regulate the expression of *PsCHS*, *PsCHI*, *PsF3H* and *PsDFR*. Meanwhile, above up-regulated structural genes may affect the metabolic balance of the branches controlled by *DFR* and *FLS* genes, thus inhibiting the biosynthesis of flavonoid components related to yellow flower formation.

### 3.6 Protein interaction between PsMYB4 and PsEGL3

**Figure 7:**
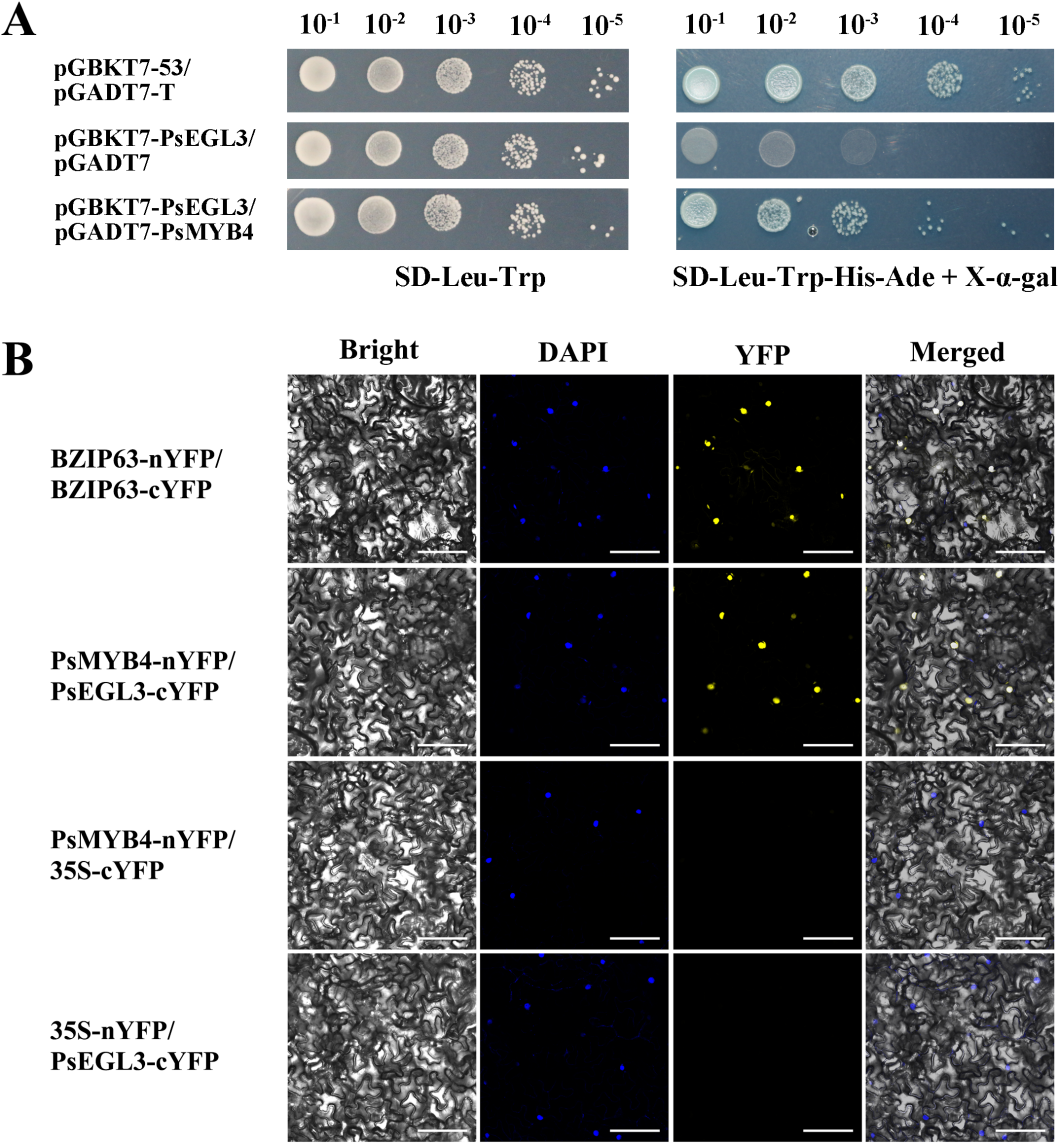
The protein interaction analysis between PsMYB4 and PsEGL3. **(A)** Y2H assay of protein interaction between PsMYB4 and PsEGL3. pGBKT7-53/pGADT7-T was positive control, pGBKT7-PsEGL3/pGADT7 was negative control. **(B)** BiFC assay confirmed the interaction between PsMYB4 and PsEGL3 in n*. benthamiana* leaves. BZIP63-nYFP/BZIP63-cYFP was positive control, PsMYB4-nYFP/35S-cYFP and 35S-nYFP/PsEGL3-cYFP were negative controls. Bright, bright field; DAPI, DAPI fluorescence; YFP, GFP fluorescence; Merged, superposition of bright field and fluorescence. Bars, 25 µm.

Considering above results, we performed Y2H and BiFC assays to confirm whether PsMYB4 interacts with PsEGL3. In the Y2H assay, the yeast cells co-transformed with pGADT7-T and pGBKT7–53 were employed as positive controls, whereas yeast cells co-transformed with pGADT7 empty vector and pGBKT7-PsEGL3 were designated as negative controls. We observed that yeast cells co-transformed with pGADT7-PsMYB4 and pGBKT7-PsEGL3 grew normally on SD-Leu-Trp-His-Ade screening medium, consistent with the positive control (**Figure 7A**), indicating an interaction between PsMYB4 and PsEGL3 in the yeast system.

The BiFC assay was performed to further validate the interaction between PsMYB4 and PsEGL3. The leaves co-transformed with BZIP63-nYFP and BZIP63-cYFP were positive controls, while leaves co-transformed with PsMYB4-nYFP/35S-cYFP or 35S-nYFP/PsEGL3-cYFP were negative controls. The fluorescence signal of YFP was observed in *N. benthamiana* leaves co-transformed with recombinant vectors PsMYB4-nYFP and PsEGL3-cYFP, as well as the positive control (**Figure 7B**). However, there was no signal in the negative control leaves (**Figure 7B**). The results obtained from BiFC and Y2H experiments provide evidence for an *in vivo* interaction between PsMYB4 and PsEGL3.

### 3.7 PsMYB4 and PsEGL3 synergistically inhibit flavonoid-related structural genes

**Figure 8:**
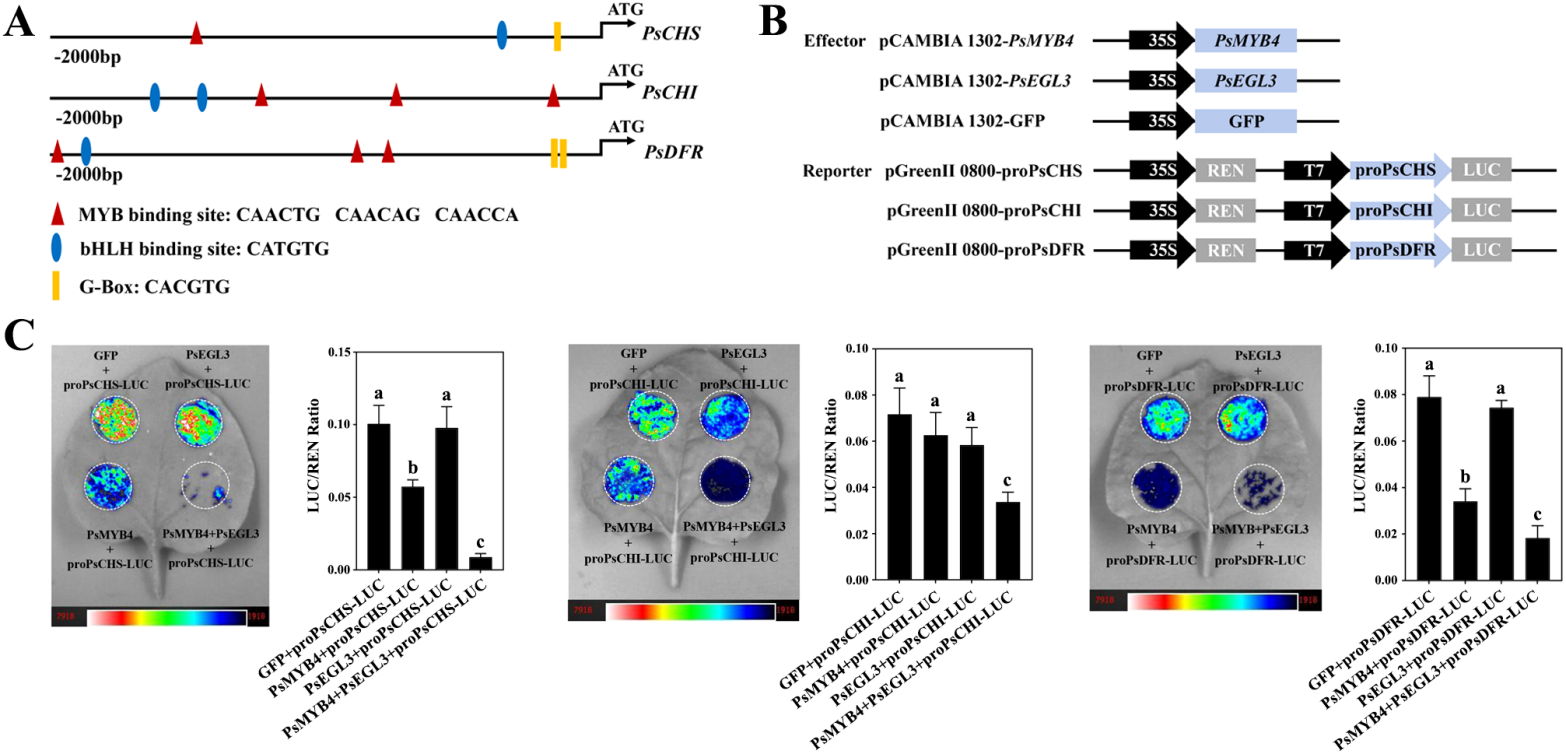
Transcription inhibition analysis of PsMYB4 and PsEGL3 against the promoters of *PsCHS*, *PsCHI*, and *PsDFR* of tree peony. **(A)** Schematic overview of *PsCHS*, *PsCHI*, and *PsDFR* promoters. **(B)** Schematic overview of the effector and reporter constructs used in the dual-luciferase assays. **(C)** Inhibition of the *PsCHS*, *PsCHI*, and *PsDFR* promoters by PsMYB4 and PsEGL3 based on dual-luciferase assays. The bar graph displays the LUC/REN ratio as a measure of normalized LUC activities. The error bars represent the SDs of six biological replicates. The samples labeled with distinct letters indicate statistically significant differences (p<0.05).

Overexpression in tobacco and VIGS in tree peony indicate that PsMYB4 and PsEGL3 may synergistically regulate flavonoid biosynthesis-related structural genes, especially *PsCHS*, *PsCHI* and *PsDFR*. To verify this hypothesis, we isolated the promoters of *PsCHS*, *PsCHI* and *PsDFR* from ‘High Noon’ petals. The identification of numerous *cis*-elements associated with MYB and bHLH TFs, such as the MYB-binding site (5’-CAACNG-3’), bHLH-binding site (5’-CATGTG-3’), and G-box (5’-CACGTG-3’) (**Figure 8A**), provides support for their potential regulatory relationships with PsMYB4 and PsEGL3.

To further clarify whether PsMYB4 and PsEGL3 directly regulate the expression of *PsCHS*, *PsCHI*, and *PsDFR* genes, a LUC transient expression assay was conducted in *N. benthamiana*. The pCAMBIA 1302 vector harboring PsMYB4 and PsEGL3 functioned as effectors, while the pGreenII0800 LUC vector carrying the promoters of *PsCHS*, *PsCHI*, and *PsDFR* served as reporters (**Figure 8B**). Consequently, both the PsMYB4/Luc-PsCHS and PsMYB4/Luc-PsDFR constructs exhibited significantly lower LUC/REN ratios compared to PsMYB4/Luc-PsCHI, indicating the inhibitory effect of PsMYB4 on the promoters of *PsCHS* and *PsDFR* (**Figure 8C**). Nevertheless, PsEGL3 had no significant inhibitory effect on *PsCHS*, *PsCHI*, and *PsDFR*. It is worth noting that the LUC/REN ratios significantly decreased when co-transformed with PsMYB4/PsEGL3/Luc-PsCHS, PsMYB4/PsEGL3/Luc-PsCHI, and PsMYB4/PsEGL3/Luc-PsDFR (**Figure 8C**), indicating that PsMYB4 and PsEGL3 synergistically inhibit the transcription of *PsCHS*, *PsCHI*, and *PsDFR*.

## 4 Discussion

Yellow cultivars are unique and precious among the nine major color systems of tree peony. The formation of their yellow color is mainly due to the abundant presence of chalcones, flavones, and flavonols, as well as the absence of anthocyanins (Li et al., 2009; Zhou et al., 2011; Shi et al., 2015; Yang et al., 2020b; Luo et al., 2021). In the present study, we also found that pure yellow tree peony cultivars did not contain anthocyanins, and the content of various flavonoid components varied among tree peony cultivars with different degrees of yellow color. Overall, among the six tree peony cultivars, the content of flavonoids including chalcone, flavones, and flavonols, gradually increased as the yellow color deepened (**Figure 2**). Interestingly, the expression levels of *PsMYB4* and *PsEGL3* were higher in the darker yellow cultivars (‘High Noon’, ‘Huangguan’ and ‘Jinge’), while lower in the purple red (‘Roufurong’), white (‘Baixueta’) and light yellow (‘Yaohuang’) cultivars (**Figure 3**), indicating that *PsMYB4* and *PsEGL3* may affect the formation of pigments such as chalcones, flavones, flavonols and anthocyanins in tree peony petals by regulating flavonoid biosynthesis pathway, and eventually lead to different flower colors. However, it is worth noting that the research on tree peony flower colors involved in this study is aimed at petal color, not including the color of spots at the petal base. As is well known, the formation of petal spots is determined by the spatiotemporal transcription of specific genes in the anthocyanin biosynthesis pathway, leading to the accumulation of specific anthocyanins in specific petal regions (Suzuki et al., 2016). In tree peony, TFs involved in the spatiotemporal regulation of petal spot formation have been explored, including PsMYB12 (Gu et al., 2018), PrMYB5 (Shi et al., 2022), and PsMYB308/PsMYBPA2/PsMYB21 (Luan et al., 2024). That is, petal background pigmentation and petal spot pigmentation may be independent (Shi et al., 2022). In this study, PsMYB4 and PsEGL3 were only responsible for petal background pigmentation of tree peony, and further research is needed to determine whether they play a role in petal spot pigmentation.

As the largest transcription factor family in plants, MYB TFs play a crucial role in regulating flavonoid biosynthesis. In addition to MYBs that play positive regulatory roles, an increasing number of MYB TFs that inhibit anthocyanin synthesis have been identified recently, including AtMYB4 in *Arabidopsis* (Wang et al., 2020), PhMYB4/27 in *Petunia hybrida* (Colquhoun et al., 2011), VvMYB4-like and VvMYBC2-L1/3 in *V. vinifera* (Cavallini et al., 2015; Pérez-Díaz et al., 2016), and MdMYB16 in *M. domestica* (Xu et al., 2017). The PsMYB4 identified in this study is a typical R2R3-MYB inhibitor, which is most closely related to AtMYB4 in the G4 subgroup of MYB TFs. Herein, overexpression of *PsMYB4* in tobacco resulted in a significant reduction in anthocyanin content, and the expression of related structural genes was inhibited to varying degrees, especially the promoters of *PsCHS* and *PsDFR* were directly targeted and inhibited by PsMYB4 (**Figures 5C, D**, **8C**). Similarly, NtMYB4, a R2R3-MYB type repressor orthologous to AtMYB4, exerted negative regulation on *NtCHS1* expression and led to reduced flavonoid accumulation (Chen et al., 2019). PqMYB4 from *P. qiui* leaves was enable to significantly lighten the seed coat color of transgeneic *Arabidopsis* by negatively regulating the expression of *AtCHS*, *AtCHI*, *AtDFR* and *AtANS* (Huo et al., 2020). However, PsMYB4 has no inhibitory effect on the expression of *PsCHI* in our study (**Figure 8C**), indicating that the MYB4 homologous gene has different regulatory patterns in different tissues of tree peony cultivars. In addition, R2R3-MYB TFs that are not part of the G4 subgroup may also play a negative regulatory role. In chrysanthemum (*Chrysanthemum* × *morifolium*), CmMYB012 is an atypical SG7 R2R3-MYB protein, while it lacks the conserved SG7 and/or SG7–2 motifs and inhibited anthocyanin biosynthesis by suppressing the expression of related structural genes (*CmCHS*, *CmDFR*, *CmANS*, and *CmUFGT*) (Zhou et al., 2021). It suggests that different subgroups of MYB TFs may be functionally redundant, but whether PsMYB4 has other regulatory functions needs to be further studied.

BHLH proteins are a family of TFs second only to MYB in plants, including 26–32 subgroups (Carretero-Paulet et al., 2010; Pires and Dolan, 2010). These bHLH proteins can either regulate the target gene alone or bind the promoter of the target gene in the form of a complex with MYB proteins. Previous studies have shown that bHLH TFs involved in regulating plant flavonoid metabolism belong to the IIIf subgroup, among which AtEGL3, AtGL3, AtTT8, and AtMYC1 are positive regulator of anthocyanin biosynthesis (Heim et al., 2023; Li H. et al., 2020; Liu et al., 2023). However, CpbHLH1, SmTT8, SmbHLH1, HLH4, and FtTT8 in the IIIf subgroup inhibit anthocyanin biosynthesis (Zhao et al., 2020; Duan et al., 2021; Hou et al., 2022; Deng et al., 2023). Similarly, PsEGL3 in this study was also clustered into the IIIf subgroup (**Figure 4B**). In addition, the significantly reduced anthocyanin content in tobacco overexpressing *PsEGL3* (**Figure 5C**), suggest that PsEGL3 may also negatively regulate anthocyanin biosynthesis. Whereas, the expression levels of *NtCHS*, *NtCHI*, *NtF3H*, *NTF3’H*, *NtFLS* and *NtDFR* in three tobacco transgenic lines were not consistent (**Figure 5E**), indicating that PsEGL3 may not be capable of independently regulating anthocyanin biosynthesis. LUC transient expression assay showed that PsEGL3 did not have a direct inhibitory effect on *PsCHS*, *PsCHI*, and *PsDFR* (**Figure 8C**), further proving that PsEGL3 cannot independently exert regulatory effects. In previous research, bHLHs primarily contribute to the assembly of MBW complexes, enhancing MYB activity rather than regulating anthocyanin synthesis alone (Liu et al., 2022). Consistent with the finding that overexpressing *RcbHLH42* and *RcEGL1* alone cannot lead to the accumulation of anthocyanin in rose petals and tabacco leaves (He et al., 2023).

In the majority of cases, R2R3-MYB and bHLH TFs, as well as WD40 proteins collaborate to form a MBW complex, which regulates structural genes participated in the flavonoid biosynthesis pathway (Zhou et al., 2011). Generally, bHLH proteins interact with both R2R3-MYB and WD40 as bridging factors, whereas the interaction between WD40 and R2R3-MYB may be absent. In our previous study, genes encoding WD40 proteins were not detected in either yellow or purple-red tree peony cultivars, which is consistent with the finding in wheat (Luo et al., 2021; Liu et al., 2023). It shows that the types and combinations of TFs regulating flavonoid biosynthesis may be different in various plant species. Two MBW complexes (RcMYB1-RcEGL1-RcTTG1, RcMYB1-RcBHLH42-RcTTG1) in rose enhanced the transcriptional activity of *RcMYB1* and late anthocyanin biosynthesis genes (He et al., 2023). A novel R2R3-MYB TF PsMYB12 in tree peony regulates organ-specific expression of *PsCHS* through the formation of a MBW complex, leading to the formation of petal blotch (Gu et al., 2018). In the present study, significantly lower LUC/REN ratios were observed for PsMYB4/PsEGL3/Luc-PsCHS, PsMYB4/PsEGL3/Luc-PsCHI, and PsMYB4/PsEGL3/Luc-PsDFR co-transformation constructs compared with PsMYB4/Luc-PsCHS, PsMYB4/Luc-PsCHI, and PsMYB4/Luc-PsDFR constructs, while PsEGL3 had no significant inhibitory effect on *PsCHS*, *PsCHI* or *PsDFR*, indicating that PsMYB4 and PsEGL3 synergistically inhibit the expression of *PsCHS*, *PsCHI*, and *PsDFR* (**Figure 8C**). However, PsMYB308 in tree peony inhibits the abundance of *PsDFR* and *PsMYBPA2* and competitively binds to PsbHLH1–3 proteins with PsMYBPA2, playing a key role in regulating anthocyanin-mediated petal blotch formation (Luan et al., 2024). In *Arabidopsis*, MYB4 (along with MYB7 and MYB32) directly interacts with EGL3, GL3, and TT8 to weaken the transcriptional activity of the MBW complexes, thereby repressing anthocyanin biosynthesis (Wang et al., 2020). Hence, there are two different regulatory modes of MYB repressors, one is the direct negative regulation of flavonoid synthesis related genes by the interaction between MYB repressors and bHLH proteins, and the other is that MYB repressors weaken the function of related MBW complexes by competitively binding to bHLH protein. The regulatory mode of PsMYB4 in this study may be the first one. In addition, the significantly increased expression levels of *PsCHS*, *PsCHI* and *PsDFR* (**Figures 6C, D**), as well as the significantly decreased contents of related chalcones, flavones and flavonols in TRV-*PsMYB4* and TRV-*PsEGL3* petals (**Figure 6B**), suggest that PsMYB4 and PsEGL3 may influence the pigmentation of tree peony yellow flowers by synergistically regulating the metabolic balance of flavonoid synthesis pathway. Interestingly, there was no significant change in *PsFLS* expression levels in both TRV-*PsMYB4* and TRV-*PsEGL3* petals (**Figures 6C, D**). It may be that the up-regulated expression of *PsCHS* and *PsDFR* leads to the increase of the total metabolic flow of flavonoid biosynthesis pathway and the metabolic flow of anthocyanin biosynthesis branch. Even if *PsFLS* maintains the original expression level, more metabolic flow still tends to the anthocyanin biosynthesis branch, thus reducing the accumulation of related chalones, flavones and flavonols. In *P. lactiflora* inner petals, a similar phenomenon was observed where the down-regulation of *PlDFR*, *PlANS*, *Pl5GT*, and *Pl3GT* resulted in the inhibition of anthocyanin biosynthesis, leading to the formation of yellow pigment (Zhao et al., 2014). In addition, we did not find the expression of *PsANS* in ‘High Noon’, which may be a reason for its anthocyanin deficiency. Hence, the flavonoid synthase genes determine the genetic basis for the formation of tree peony yellow flowers, especially the competition mechanism between *PsFLS* and *PsDFR*.

## 5 Conclusion

**Figure 9:**
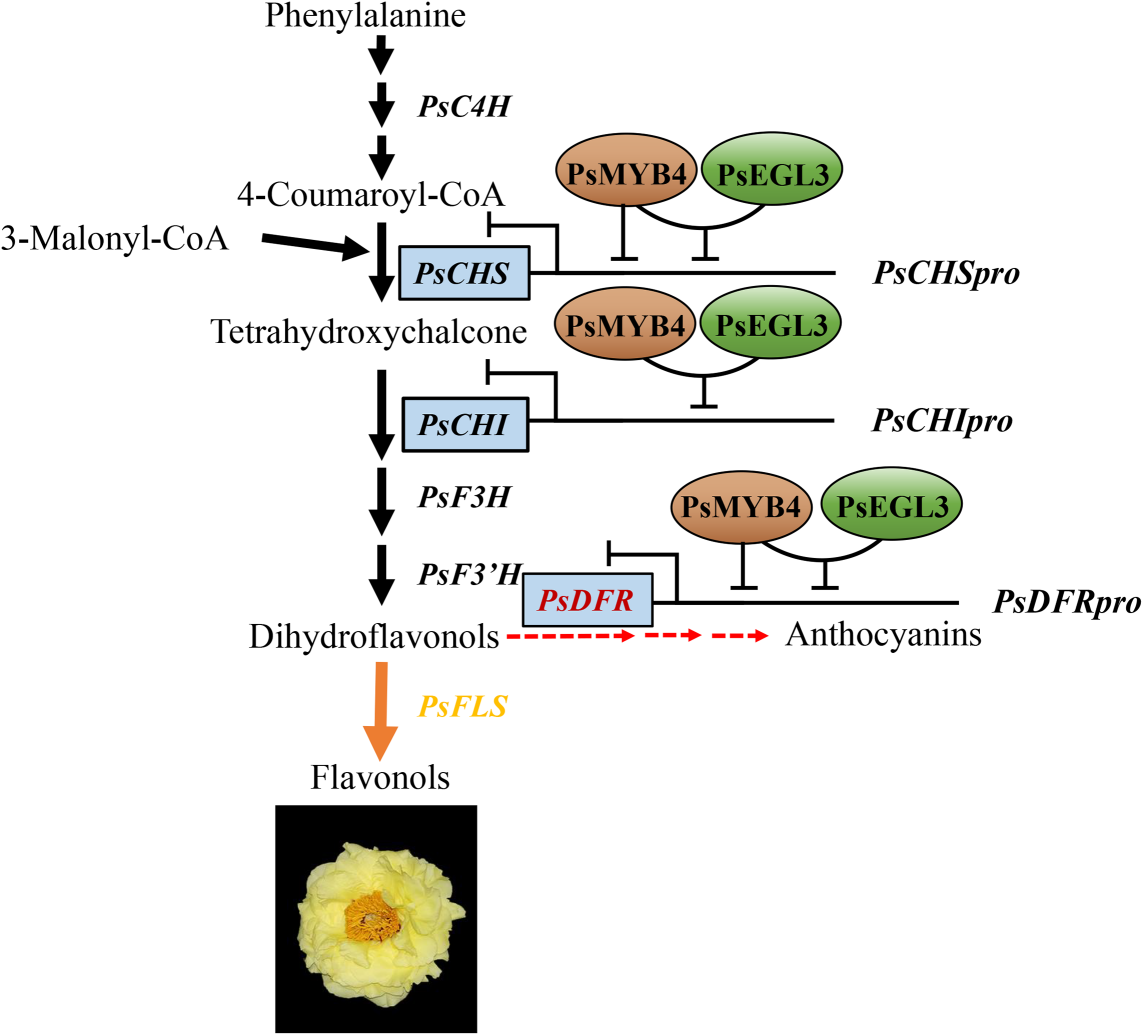
A working model of PsMYB4 and PsEGL3 involved in regulating the formation of tree peony yellow flowers. PsMYB4 has inhibitory effects on the promoters of *PsCHS* and *PsDFR*, but not on the promoter of *PsCHI*, while PsEGL3 has no inhibitory effects on the promoters of *PsCHS*, *PsCHI*, and *PsDFR*. PsMYB4 and PsEGL3 can synergistically inhibit the expression of *PsCHS*, *PsCHI*, and *PsDFR* through interaction, thereby preventing the metabolic flow on the anthocyanin synthesis branch, leading to a trend towards the flavonol synthesis branch and ultimately promoting the formation of tree peony yellow flowers.

In summary, PsMYB4, belonging to the R2R3-MYB repressor, was identified to inhibit anthocyanin biosynthesis. PsEGL3, which was clustered into the subgroup IIIf of bHLH TF family, assisted PsMYB4 in negatively regulating the expression of *PsCHS*, *PsCHI*, and *PsDFR*, thereby inhibiting the anthocyanin biosynthesis branch in tree peony yellow flowers (**Figure 9**). In combination with our previous study, PsMYB111 promotes the synthesis of chalcone and flavonol by positively regulating the expression of *PsCHS* and *PsFLS*, further exacerbating the metabolic imbalance between the flavonol synthesis branch and the anthocyanin synthesis branch, ultimately leading to a complete shift in metabolic flux towards flavonol synthesis and promoting the formation of tree peony yellow flowers (Luo et al., 2021). These findings provide comprehensive insights for the regulatory mechanism of tree peony yellow flower formation and may contribute to the production of novel yellow tree peony cultivars with high ornamental value.

## Data Availability

The original contributions presented in the study are included in the article/**Supplementary Material**. Further inquiries can be directed to the corresponding authors.
