# Supplementary material for: Interaction of PsMYB4 with PsEGL3 inhibits anthocyanin biosynthesis in tree peony yellow flowers
Source: Front Plant Sci. 2025 Jun 23;16:1595014. doi: 10.3389/fpls.2025.1595014 (PMC12230082; doi:10.3389/fpls.2025.1595014)
Supplement: Supplementary file 2 [file Table1.doc]

| **Supplementary Table 1 Primers used for qRT-PCR in tree peony.** | | |
| --- | --- | --- |
| **Primers** | **Primer Sequences (5'-3')** | **Description** |
| *PsUbiquitin*-F | GACCTATACCAAGCCGAAG | qRT-PCR for tree peony |
| *PsUbiquitin*-R | CGTTCCAGCACCACAATC | qRT-PCR for tree peony |
| *PsC4H*-F | GTAATCTGGTGGTGGTTTCGT | qRT-PCR for tree peony |
| *PsC4H*-R | TCGTAAAGAAAGGAACCGTCA | qRT-PCR for tree peony |
| *PsCHS*-F | ACTACCAACTCACCAAACTCC | qRT-PCR for tree peony |
| *PsCHS*-R | CGACCAAAGAATCCAAATGAG | qRT-PCR for tree peony |
| *PsCHI*-F | CGCAGGAGTAAGAGGTTTGGA | qRT-PCR for tree peony |
| *PsCHI*-R | CTCTGAATATTGTTGGCCCGT | qRT-PCR for tree peony |
| *PsF3H*-F | AAAACCCTTCAATCCAGCTTC | qRT-PCR for tree peony |
| *PsF3H*-R | AAATCCCCCAGTCTTCACATG | qRT-PCR for tree peony |
| *PsF3’H*-F | TTTTGTGATACTGCCCCCTCT | qRT-PCR for tree peony |
| *PsF3’H*-R | TATTTAGTGCAAAGATGGCCC | qRT-PCR for tree peony |
| *PsFLS*-F | CACTCTCAACCAAAACAATCC | qRT-PCR for tree peony |
| *PsFLS*-R | CACTCTCAACCAAAACAATCC | qRT-PCR for tree peony |
| *PsDFR*-F | GTTAATGGACTCTGGGTTTGA | qRT-PCR for tree peony |
| *PsDFR*-R | ATCCCTTTCCTCGGCATGTTT | qRT-PCR for tree peony |
| *PsMYB4*-F | GGCTAATCAAAGAGGAGGTCA | qRT-PCR for tree peony |
| *PsMYB4*-R | ACTGCTACTTCCACCACCATT | qRT-PCR for tree peony |
| *PsEGL3*-F | AAAAGAATTGACGAGGACCAG | qRT-PCR for tree peony |
| *PsEGL3*-R | AGCTCCACCATTTATGCCATC | qRT-PCR for tree peony |

Supplementary Table 2 Homologous MYBs from other plant species and their login numbers in the NCBI database.

| Gene Name | Gene ID | Gene Name | Gene ID |
| --- | --- | --- | --- |
| EgMYB1 | CAE09058.1 | AtMYB14 | AAS10045.1 |
| AsMYB4 | AYA44248.1 | OsMYB4 | CAA72217.1 |
| AtMYB4 | AAC83582 | PtMYB134 | ACR83705 |
| CmMYB1 | AEO27497.1 | VvMYB5a | AAS68190 |
| ZmMYB31 | NP_001105949 | VvMYB5b | Q58QD0 |
| FtMYB3 | AEC32978.1 | MdMYB110a | JN711473 |
| PgMYB | AIO09658.1 | MdMYB10 | ACQ45201 |
| AtMYB32 | NP_195225 | MtLAP1 | FJ199998 |
| AtMYB7 | NP_179263 | VvMYBA1 | BAD18977 |
| ZmMYB42 | NP_001106009 | VvMYBA2 | BAD18978.1 |
| PvMYB4a | AEM17348 | AtPAP1 | AAG42001 |
| VvMYBF1 | FJ948477 | AtPAP2 | AAG42002 |
| AtMYB11 | NP_191820 | SmMYB1 | KT259043 |
| AtMYB111 | NP_199744 | NtAN2 | ACO52470.1 |
| EgMYB2 | CAE09057.1 | AtCPC | NP_182164 |
| NaMYB4 | OIT38711.1 | PhMYBx | AHX24371 |
| EjMYB1 | AID56313.1 | VuMYBR3 | KT186105 |

Supplementary Table 3 Homologous bHLHs from other plant species and their login numbers in the NCBI database.

| Gene Name | Gene ID | Gene Name | Gene ID |
| --- | --- | --- | --- |
| TrAN1 | AIT76559 | PhJAF13 | AAC39455 |
| MtTT8 | KM892777 | AtGL3 | NP_680372 |
| LjTT8 | BAH28881 | AtEGL3 | Q9CAD0 |
| VvMYC1 | ACC68685 | VvMYCA1 | ABM92332 |
| PpbHLH3 | AIE57508 | CsMYC2 | ABR68793.1 |
| MdbHLH3 | ADL36597 | MdbHLH33 | ABB84474 |
| PhAN1 | AAG25927.1 | AtbHLH061 | AAM10950 |
| AtTT8 | Q9FT81 | AtbHLH116 | AAL84972 |
| BrTT8 | AEA03281.1 | AtDYT1 | O81900 |
| AcB2 | AUG71567 | AtbHLH021 | NP_179283 |
| LhbHLH2 | BAE20058 | AtbHLH027 | AAS79544 |
| ZmIN1 | AAB03841 | AtbHLH035 | NP_974948 |
| OsRc | ABB17166 | AtJAM2 | Q9LNJ5 |
| ZmR | P13027 | AtbHLH017 | AAM19778 |
| MtEGL3 | Medtr8g098275.1 | AtMYC2 | Q39204 |
| TrJAF13 | AIT76563 | AtbHLH028 | AAL55721 |
| CpbHLH1 | QKL20124.1 |  |  |

| **Supplementary Table 4 Primers used for subcellular localization.** | | |
| --- | --- | --- |
| **Primers** | **Primer Sequences (5'-3')** | **Description** |
| *PsMYB4*-F | ACACGGGGGACTCTTGACATGGGAAGGTCTCCTTGTTGT | In-fusion cloning |
| *PsMYB4*-R | AAGTTCTTCTCCTTTACTCTTCATCTCCAATCTTCTGTA | In-fusion cloning |
| *PsEGL3*-F | ACACGGGGGACTCTTGACATGGCAACTGGGCTCCAAATT | In-fusion cloning |
| *PsEGL3*-R | AAGTTCTTCTCCTTTACTACACTTGTGAATAACTCTCTG | In-fusion cloning |

| **Supplementary Table 5 Primers used for qRT-PCR in transgenic tobacco.** | | |
| --- | --- | --- |
| **Primers** | **Primer Sequences (5'-3')** | **Description** |
| *NtTubA1*-F | CTCCTATGCTCCTGTCATTTC | qRT-PCR for transgenic tobacco |
| *NtTubA1*-R | GGCGAGGATCACACTTAAC | qRT-PCR for transgenic tobacco |
| *NtC4H*-F | ACAACAGAGAAAGGCGGGCAA | qRT-PCR for transgenic tobacco |
| *NtC4H*-R | AGGGGCTTTACATGACACCAA | qRT-PCR for transgenic tobacco |
| *NtCHS*-F | TGACACCCACTTGGATAGTTTAG | qRT-PCR for transgenic tobacco |
| *NtCHS*-R | CGACCTCTGGAATTGGATCAG | qRT-PCR for transgenic tobacco |
| *NtCHI*-F | CTTTTCTCGCCGCTAAATG | qRT-PCR for transgenic tobacco |
| *NtCHI*-R | TTTCTGCCACCTTCTCTG | qRT-PCR for transgenic tobacco |
| *NtF3H*-F | CAAGGCATGTGTGGATATGG | qRT-PCR for transgenic tobacco |
| *NtF3H*-R | TGTGTCGTTTCAGTCCAAGG | qRT-PCR for transgenic tobacco |
| *NtF3’H*-F | AGGCTCAACACTTCTCGT | qRT-PCR for transgenic tobacco |
| *NtF3’H*-R | CATCAACTTTGGGCTTCT | qRT-PCR for transgenic tobacco |
| *NtFLS*-F | GAACTTGAAGGGAAAAGGGGTTG | qRT-PCR for transgenic tobacco |
| *NtFLS*-R | GTAGGAGGGAGGATTTTTAGGCC | qRT-PCR for transgenic tobacco |
| *NtDFR*-F | AACCAACAGTCAGGGGAATG | qRT-PCR for transgenic tobacco |
| *NtDFR*-R | TTGGACATCGACAGTTCCAG | qRT-PCR for transgenic tobacco |

| **Supplementary Table 6 Primers used for virus-induced gene silencing in tree peony.** | | |
| --- | --- | --- |
| **Primers** | **Primer Sequences (5'-3')** | **Description** |
| TRV-*PsMYB4*-F | CCCATATGGTCGACCTGCAGGGCTAATCAAAGAGGAGGTCA | In-fusion cloning |
| TRV-*PsMYB4*-R | GGACATGCCCGGGCCTCGAGTCCATCTTTACTTCATCTCCA | In-fusion cloning |
| TRV-*PsEGL3*-F | CCCATATGGTCGACCTGCAGGACGAGGACCAGGCTTGTTTT | In-fusion cloning |
| TRV-*PsEGL3*-R | GGACATGCCCGGGCCTCGAGGTCCTTTACCTCTTCAATCTT | In-fusion cloning |
| TRV-*PsMYB4*-F | GAGATTACCAGGCAGAACAGA | qRT-PCR |
| TRV-*PsMYB4*-R | AACCCAGCAGAACCAAAGACG | qRT-PCR |
| TRV-*PsEGL3*-F | ACATCTGATAACTACGGCAAC | qRT-PCR |
| TRV-*PsEGL3*-R | ACCACAGCATCTTTCTCCATT | qRT-PCR |
| TRV-*PsC4H*-F | GTAATCTGGTGGTGGTTTCGT | qRT-PCR |
| TRV-*PsC4H*-R | TCGTAAAGAAAGGAACCGTCA | qRT-PCR |
| TRV-*PsCHS*-F | ACTACCAACTCACCAAACTCC | qRT-PCR |
| TRV-*PsCHS*-R | CGACCAAAGAATCCAAATGAG | qRT-PCR |
| TRV-*PsCHI*-F | CGCAGGAGTAAGAGGTTTGGA | qRT-PCR |
| TRV-*PsCHI*-R | CTCTGAATATTGTTGGCCCGT | qRT-PCR |
| TRV-*PsF3H*-F | AAAACCCTTCAATCCAGCTTC | qRT-PCR |
| TRV-*PsF3H*-R | AAATCCCCCAGTCTTCACATG | qRT-PCR |
| TRV-*PsF3’H*-F | TTTTGTGATACTGCCCCCTCT | qRT-PCR |
| TRV-*PsF3’H*-R | TATTTAGTGCAAAGATGGCCC | qRT-PCR |
| TRV-*PsFLS*-F | CACTCTCAACCAAAACAATCC | qRT-PCR |
| TRV-*PsFLS*-R | CACTCTCAACCAAAACAATCC | qRT-PCR |
| TRV-*PsDFR*-F | GTTAATGGACTCTGGGTTTGA | qRT-PCR |
| TRV-*PsDFR*-R | ATCCCTTTCCTCGGCATGTTT | qRT-PCR |

| **Supplementary Table 7 Primers used for yeast two-hybrid assay.** | | |
| --- | --- | --- |
| **Primers** | **Primer Sequences (5'-3')** | **Description** |
| AD-PsMYB4-F | CCGGAATTCATGGGAAGGTCTCCTTGTTGT | In-fusion cloning |
| AD-PsMYB4-R | TCCCCCGGGTTACTTCATCTCCAATCTTCT | In-fusion cloning |
| BD-PsEGL3-F | CATGCCATGGATGGCAACTGGGCTCCAAAT | In-fusion cloning |
| BD-PsEGL3-R | TCCCCCGGGCTAACACTTGTGAATAACTCT | In-fusion cloning |

| **Supplementary Table 8 Primers used for bimolecular fluorescence complementation assay.** | | |
| --- | --- | --- |
| **Primers** | **Primer Sequences (5'-3')** | **Description** |
| NYFP-PsMYB4-F | CAGGCCTGGCGCGCCACTAGTGGATCCATGGGAAGGTCTCCTTG | In-fusion cloning |
| NYFP-PsMYB4-R | GCTCCATCCCGGGAGCGGTACCCTCGAGCTTCATCTCCAATCTTCTGTA | In-fusion cloning |
| CYFP-PsEGL3-F | CAGGCCTGGCGCGCCACTAGTGGATCCATGGCAACTGGGCTCC | In-fusion cloning |
| CYFP-PsEGL3-R | GTACATCCCGGGAGCGGTACCCTCGAGACACTTGTGAATAACTCTCTGAAG | In-fusion cloning |

| **Supplementary Table 9** **Primers used for luciferase reporter assay.** | | |
| --- | --- | --- |
| **Primers** | **Primer Sequences (5'-3')** | **Description** |
| proPsCHS-F | GTACCGGGCCCCCCCTCGAGGTCGACAGTATGTATGAAAATATTATTTCTTATATTAAC | Dual-luciferase assay |
| proPsCHS-R | GGCTGCAGGAATTCGATATCAAGCTTTTGTGTGTCT GGGCTCGGTT TTACACTGAG | Dual-luciferase assay |
| proPsCHI-F | GTACCGGGCCCCCCCTCGAGGTCGACAGCTATGATTATATGGTTTGTGCTTTTAAGTCC | Dual-luciferase assay |
| proPsCHI-R | GGCTGCAGGAATTCGATATCAAGCTTTATTTTGTGTCTGGGTCTGGAACTC | Dual-luciferase assay |
| proPsDFR-F | GTACCGGGCCCCCCCTCGAGGTCGACAGCTTCTGCAACCACAAGATCACCGACCGTCTT | Dual-luciferase assay |
| ProPsDFR-R | GGCTGCAGGAATTCGATATCAAGCTTTTGCTTTTGT TTTTTAACCACGATATGAGGA | Dual-luciferase assay |
